# Detection of Genetic Variants in Thai Population by Trio-Based Whole-Genome Sequencing Study

**DOI:** 10.3390/biology14030301

**Published:** 2025-03-17

**Authors:** Patcharin Boonin, Sommon Klumsathian, Nareenart Iemwimangsa, Insee Sensorn, Angkana Charoenyingwatana, Wasun Chantratita, Takol Chareonsirisuthigul

**Affiliations:** 1 Department of Pathology, Faculty of Medicine Ramathibodi Hospital, Mahidol University, Bangkok 10400, Thailand; patcharin.bon@student.mahidol.edu; 2 Center for Medical Genomics, Faculty of Medicine, Ramathibodi Hospital, Mahidol University, Bangkok 10400, Thailand; sommon4@gmail.com (S.K.); nareenart.iem@mahidol.ac.th (N.I.); insee.sen@mahidol.ac.th (I.S.); angkana.chr@mahidol.ac.th (A.C.); wasun.cha@mahidol.ac.th (W.C.)

**Keywords:** family-based whole-genome sequencing, Thai population, WGS, Trio-Based whole-genome sequencing

## Abstract

This trio-based whole-genome sequencing (WGS) study enhances the accuracy of variant detection by leveraging parental genotypes, which facilitates the identification of de novo mutations and population-specific variants. Nonetheless, the comprehensive genetic variation data of the Thai population remain limited, posing challenges to advancing personalized medicine and population-based screening strategies. We establish the genetic variation information of a healthy Thai population by analyzing the sequences of 40 trios, yielding 120 whole genomes (excluding offspring). The resulting dataset encompasses 20.2 million variants, including 1.1 million novel and 19.1 million known variants. Within this dataset, we identify 169 pathogenic variants, of which 56 are classified as rare and 87 are absent from the ClinVar database as of version 2023. These pathogenic variants, particularly the rare and de novo mutations, will likely be of significant interest for genetic association studies. Notably, one pathogenic variant linked to a de novo mutation is found in the *SF3B2* gene, which is associated with craniofacial microsomia. With its innovative methodology and comprehensive dataset, our trio-based whole-genome sequencing study provides an invaluable representation of the genetic variations in the Thai population. These data provide a critical foundation for further analyses of the pathogenic variants related to human disease phenotypes in genetic association studies.

## Simple Summary

This study analyzes 120 whole genomes and 40 trio sequences, revealing significant genetic variations within a healthy Thai population. Understanding these genetic differences is essential for designing clinical research and implementing effective population screenings. Insights into pathogenic variants can enhance screening tests to prevent diseases among Thai individuals. For instance, mutations in the *HBB* and *HBA2* genes are linked to thalassemia, while *ATP7B* mutations cause Wilson’s disease. Additionally, genes like *PDE4DIP*, *UBXN11*, and *AXIN1* are associated with hepatocellular carcinoma, and a de novo mutation in the *SF3B2* gene relates to craniofacial microsomia. These findings provide a foundation for advancing research and improving Thailand’s genetic screening and disease prevention.

## 1. Introduction

The human genome project (HGP), completed in April 2003, provided a complete human genome reference sequence, enabling comprehensive studies of genetic variations. Following the completion of the HGP, large-scale whole-genome sequencing (WGS) studies have been conducted to create genetic variation databases across various populations. This work has facilitated advancements in personalized medicine, disease prediction, and genetic screening in countries like the United Kingdom, Japan, the Netherlands, Vietnam, and Singapore. Global initiatives, such as the 1000 Genomes Project and the Genome Aggregation Database (gnomAD), have also been established to document human genetic diversity, offering crucial insights into rare and population-specific variants [1]. The Genome of the Netherlands (GoNL) project further enhanced this effort by employing a family-based design, improving the accuracy of variant detection and identifying de novo mutations by leveraging genetic inheritance patterns within families [2].

In Southeast Asia, however, genetic diversity is still underrepresented in global genomic databases. Recent efforts have focused on developing population-specific references, such as the Vietnamese Genome Project, which sequenced WGS and whole-exome sequencing (WES) data from healthy Kinh individuals [3]. The SG10K Project in Singapore also analyzed the genomic diversity among the Chinese, Malay, and Indian populations.

The trio design in whole-genome sequencing allows for the characterizing of new or de novo mutations within a population. De novo mutations (DNMs) are genetic changes that occur for the first time in a family member due to a variant in a germ cell (egg or sperm) during gametogenesis in one of the parents or because a mutation arises in the fertilized egg itself during early embryogenesis or post-zygotically [4]. The essential characteristics of whole-genome trio sequencing can reduce false-positive variant calls in Mendelian segregation by comparing genomic data from both parents [5]. One distinct advantage of trio data is identifying de novo mutation events by detecting new variants in the offspring that are absent in either parent [2,6].

Exploring de novo mutations between generations is vital for investigating genetic diseases and understanding the timing of these mutations in human evolution [7,8]. DNMs play a significant role in both rare and common neurodevelopmental disorders, including intellectual disabilities, autism, and schizophrenia [4]. However, there remain uncertainties regarding the actual rate of de novo events and the effects of paternal age [9].

Standard whole-genome sequencing conducted at 15–30× coverage with a trio design can detect de novo mutations across any gene unbiasedly. This technique is also more sensitive to detecting structural variations. In terms of somatic mutations, the detection of de novo mutations is possible if they are present in more than 20% of cells [10].

Given the significance of the genetic variations within specific races or geographic origins for clinical research, medical planning, and population screening. There was a necessity to construct a genome reference panel based on trio-based whole-genome sequencing studies of the genetic variations in the Thai population.

## 2. Materials and Methods

### 2.1. Sample Collection

This project initiated a pilot study involving the healthy Thai population, using the whole-genome sequence data from the trio-based sequencing of 40 trios, which included 120 volunteers. Each trio consists of two parents and one offspring. The inclusion criteria for the volunteers were as follows: (i) participants must be over 18 years of age and possess healthy physical characteristics; (ii) both generations of volunteer parents and their spouses must have been born in Thailand; and (iii) volunteers, their spouses, and their offspring must be willing to sign a consent form.

The exclusion criteria included the following: (i) if the volunteer’s family was not born in Thailand; (ii) if the volunteer is found to have significant health problems during the study; and (iii) if the volunteer decides to withdraw from the project.

The volunteers provided approximately 6 mL of blood collected in EDTA tubes. Two hundred microliters of blood was processed using a QuickGene DNA Whole Blood Kit from Kurabo Industries Ltd. (Tokyo, Japan). The concentration of genomic DNA was measured with a Qubit fluorometer from Thermo Fisher Scientific (Waltham, MA, USA), and the quality was evaluated via agarose gel electrophoresis at BGI Genomics (Shenzhen, China).

This study protocol, COA. MURA2023/304, received approval from the committee on human rights-related research involving human subjects at the Faculty of Medicine, Ramathibodi Hospital, Mahidol University.

### 2.2. Whole-Genome Sequencing

Genomic DNA (gDNA) samples were sequenced on the DNBseq platform for whole-genome sequencing, specifically using paired-end 100 bp sequencing at BGI Genomics.

DNA fragments ranging from 100 to 300 base pairs were selected and modified at the 3′ end through A-tailing for library preparation. Then, dTTP-tailed adapters were ligated to both ends of the DNA fragments. The ligated products underwent PCR amplification, followed by purification and heat denaturation.

A specialized molecule reverse complementary to the target sequence was then ligated using DNA ligase and digested with exonuclease. This process resulted in the forming of a single-stranded circular DNA library ready for sequencing.

After sequencing, the data underwent a cleaning process that included adapter removal and the filtering of low-quality reads to ensure high-quality results for downstream analysis at BGI Genomics.

### 2.3. Variant Calling and Annotation

The fastp (version 0.20.0) filters out low-quality reads and bases. Subsequent bioinformatics analyses were performed on these qualified data. A total of 240 FastQ files were analyzed using the FastQC software version 0.11.9, and a comprehensive quality assessment report was generated by merging the results with MultiQC.

Genomic variant call format (gVCF) files from each sample were used to call variants employing the Haplotyper in the Sentieon software (version 201808.08 from Sentieon^®^, San Jose, CA, USA). These variants were aligned with the human reference genome hg19 (GRCh37), which includes the decoy sequences referred to as hs37d5. The gVCFs from the trio were then combined using the GVCFtyper in the Sentieon software.

The 40 variant call format (VCF) files from the joint calling step were filtered against the germline variant truth sets using the Variant Quality Score Recalibration (VQSR) in the Sentieon software. The truth sets for the SNP and INDEL analyses included data from the HapMap and 1000 Genomes Projects.

The final step in the family-based whole-genome sequencing study involved genotype refinement using the Genome Analysis Toolkit (GATK) (package version 4.0.12.0). The bioinformatics tools used in the genotype refinement pipeline included the CalculateGenotypePosteriors, VariantFiltration, and VariantAnnotator (PossibleDeNovo). Additionally, the VarSeq^®^ software version 2.2.1 (Golden Helix^®^, Bozeman, MT, USA) was employed for variant filtration and annotation with default parameters.

## 3. Results

### 3.1. Demographic Data

The geographical distribution of the participants is illustrated in Figure S1. A total of 120 participants from 40 families in Thailand were included in the study. The information gathered included the province of birth, as well as the country of birth of their parents, along with relevant health data. The median maternal age at the time of the blood draw was 57 years, ranging from 43 to 70 years, while the median paternal age was 62, with a range of 45 to 80 years. The median age of the offspring was 27 years, ranging from 18 to 37 years (see Table 1). Among the 40 offspring, there were 26 females and 14 males. It is important to note that all participants were healthy volunteers.

### 3.2. Whole-Genome Sequencing Quality

All 240 FastQ files exhibited excellent quality, with a Phred score of over 30 (see Figure S2). The average read depth was 45.2×, with a minimum of 32.1× and a maximum of 51.8× (refer to Figure S3). The ratio of transitions to transversions also averaged 2.016 (see Figure S4).

### 3.3. Whole-Genome Sequencing Variant Detection in a Healthy Thai Population

Whole-genome sequencing variant detection was conducted using 80 samples from the Thai individuals (40 maternal and 40 paternal). All variants were annotated with the VarSeq^®^ software version 2.2.1, filtering for genotype qualities (GQ) greater than 20. The dbSNP155 database identified a total of 20,217,302 variants, categorized into 19,112,577 known variants (94.54%) and 1,104,725 novel variants (5.46%), which were not present in the dbSNP155 database. Among the known variants, the composition included 62.1% single nucleotide variants (SNVs), 29.9% insertions and deletions (INDELs), 1.5% deletions (DELs), 1.3% insertions (INSs), and 0.0007% multi-nucleotide variants (MNVs) (see Table 2).

To determine the allele frequency (AF) of the whole-genome sequencing variants in the 80 Thai individuals, we categorized the variants into three groups: common variants (greater than 0.05), low-frequency variants (between 0.01 and 0.05), and rare variants (less than 0.01) [11] The analysis utilized data from the 1000 Genomes Project database (1KGP) [12]. Out of the 80 healthy Thai individuals whose whole-genome sequences were analyzed, we found that 15.08% of the variants were rare, 7.03% were low-frequency, and 37.57% were common. Additionally, 40.32% of the variants were missing, meaning they were not identified in the 1KGP database. Almost all of the variants (98.09%) were classified as intergenic or intron in the RefSeq database (Table 3).

ClinVar is a database that provides interpretations of genetic variants concerning their clinical significance. It categorizes these variants into five classifications: Pathogenic, Likely Pathogenic, Uncertain Significance, Likely Benign, and Benign [13]. The ClinVar database version from 2023 identified 119,933 disease-associated variations (see Table 4). Among these, 169 were classified as pathogenic variants, of which 56 were considered rare. Additionally, 89 variants were not included in the 143 pathogenic mutations, encompassing approximately 100 genes linked to conditions such as cancer, blood diseases, cardiovascular disorders, immunodeficiencies, metabolic issues, reproductive system problems, and others (see Table S1).

The information indicates that cancer is one of the disorders with the highest number of pathogenic variants, followed by conditions related to metabolism, blood disorders, the reproductive system, immunodeficiencies, and cardiovascular issues. This underscores the complexity of the genetic factors in these health conditions.

The 1KGP database was constructed using the data from 14 populations; however, it did not include the Thai population [12]. Table 5 presents the variants with allele frequencies of less than 0.01 that are missing in the 1KGP database but have allele frequencies of greater than 0.05 in 80 whole-genome sequencing databases from healthy Thai individuals.

Twenty pathogenic mutations associated with various conditions, including hepatocellular carcinoma, lung cancer, thalassemia, serotonin transporter activity, spinocerebellar ataxia, and fragile X tremor/ataxia syndrome, were identified in the ClinVar database. Among these twenty mutations, only one is classified as a rare variant in the 1KGP, while the others have no corresponding data in that database.

Another notable finding is related to the frequency of the *HBB* gene mutation associated with the hemoglobin E/beta thalassemia disease. In the 1KGP database, the frequency of this mutation is 0.0028, whereas it is 0.0625 among the 80 healthy Thai volunteers, indicating a difference of 22 times. This mutation is identified as a missense variant (NM_000518.4:c.79G>A) in the *HBB* gene.

### 3.4. De Novo Mutation of a Healthy Thai Population Using Trio Whole-Genome Sequencing

All 40 paternal-mother-offspring Thai trios were identified as de novo variants in the offspring that were not present in either of the parents using PossibleDeNovos in the GATK 4.0.12.0. The mean of hiconfdenovo or high confidence possible de novo mutation (all the trio samples with the genotype quality more significant than 20 indicate that the genotype may be present at the site) of the trio was 675.35 (358–904: SD = 138.01). However, the parent’s age was associated with some de novo mutations in their offspring, as shown in Figure S5. This association suggests that an advanced parental age may be a risk factor for de novo mutations in their offspring, a finding that warrants further investigation. There is no significant difference when analyzing regression statistics. Forty trios in Thailand show an average paternal age of 33.6 and a mother age of 29.9.

All hiconfdenovo of the 40 Thai trios identified 19,710 variants, divided into 14,503 (73.58%) known variants and 5207 (26.42%) novel variants in the dbSNP155 database. Most de novo variants were intergenic (65.50%), and intron variants (32.74%) in the RefSeq database, as shown in Table 6, and the ClinVar database identified one as pathogenic, six as uncertain significance, four as likely benign, and 18 as benign. The pathogenic variant in the de novo mutation is craniofacial microsomia, an abnormality in which a part of the face does not develop and grow normally (Table 7).

## 4. Discussion

Geographically, Thailand covers an area of about 514,000 km^2^, with a population of nearly 66.2 million as of 2021 [14]. It shares borders with several countries, including Myanmar, Laos, Cambodia, and Malaysia. Historical evidence indicates that various groups have migrated to Thailand [15], contributing to the genetic diversity of its population. While whole-genome sequencing has been studied in Southeast Asian countries such as Vietnam and Singapore, trio-based studies in Thailand are currently lacking. In Singapore, whole-genome sequencing has been used to investigate the demographics and the historical evolution of Asian populations [16]. Conversely, trio-based whole-genome sequencing studies have been conducted in the Netherlands, utilizing biological sample collections based on population and patient data to investigate de novo mutations [17].

Our pilot study on trio-based whole-genome sequencing represents a unique and significant contribution to understanding a healthy Thai population. Notably, a trio design study on the Dutch population achieved a median depth of approximately 15× in 2015 [17]. Our study, however, has exceeded this benchmark, reaching more than 30× read coverage, which represents a substantial enhancement in data quality. This high read coverage is not merely a statistic; it is a pivotal achievement that ensures a comprehensive and accurate representation of the genome, thereby increasing the reliability of our findings.

The genotype refinement workflow employed in our project facilitated variant discovery, serving as a crucial tool for analyzing families that require the precise identification of individual genotypes. This workflow is essential for improving the accuracy of genotype calls while filtering out unreliable data. Furthermore, the high-coverage whole-genome sequencing (WGS) data generated in this pilot project enables the efficient collection of genetic variation data from a healthy Thai population. The information obtained regarding the pathogenic variants in the Thai population has the potential to significantly influence the development of screening tests aimed at disease prevention. For example, the *HBB* and *HBA2* genes are associated with thalassemia, the *ATP7B* gene is linked to Wilson’s disease, and a specific set of genes is related to hepatocellular carcinoma. Our study involved healthy Thai volunteers, and no phenotypic data were collected. While we identified the pathogenic variants associated with certain diseases, their clinical implications in this cohort remain unknown. These findings provide a foundation for future research that can compare genetic variations with phenotypic data and aid in developing population-specific screening tests for early disease detection in Thailand.

In our analysis of 40 trio whole-genome sequences from Thai participants, we utilized the VariantAnnotator PossibleDeNovo tool in the GATK version 4.0.12.0, part of the genotype refinement workflow for identifying germline short variants. We also developed an algorithm known as PhaseByTransmission (PBT) [18]. Previous studies have demonstrated that mutation rates are influenced by paternal age [19], a pattern also observed in our project. Following earlier research that identified the mutations in the *SF3B2* gene as a significant factor in craniofacial microsomia (CFM), our study found that *SF3B2* mutations are the most prevalent genetic cause of CFM, accounting for approximately 3% of sporadic cases and about 25% of familial instances. This finding emphasizes the importance of the *SF3B2* gene in improving our understanding of and potentially treating CFM [20]. Further research involving patients with genetic conditions may enhance our ability to distinguish specific pathogenic de novo mutations from the baseline and identify trusted related de novo mutations based on these findings [21].

## 5. Conclusions

This study, based on the whole-genome sequencing of a trio, highlights significant genetic variations within the Thai population. Understanding this genetic diversity is crucial for accurately interpreting the results of whole-exome and whole-genome sequencing in Thai patients. It is essential to investigate the genes influencing a population’s susceptibility to certain diseases to develop appropriate tests for the local population. These data can further analyze the pathogenic variants associated with human disease phenotypes in genetic association studies.

## Figures and Tables

**Table 1:** The demographic data of the participants at the blood draw.

Participants	Female	Male	Median Age (Year)
Maternal (*n* = 40)	40		57.3 (43–70)
Parental (*n* = 40)		40	61.0 (45–80)
Offspring (*n* = 40)	26	14	27.3 (18–37)

**Table 2:** Detection of genomic variants in Thai individuals using whole-genome sequencing, as referenced in the dbSNP155 database.

Databases	No. of Variants	Type of Variants
Total variants	20,217,302	
Novel variants	1,104,725 (5.46%)	
Known variants	19,112,577 (94.54%)	SNV = 62.1% INDEL = 29.9% DEL = 1.5% INS = 1.3% MNV = 0.0007%

**Table 3:** Whole-genome sequencing variant detection of Thai individuals in the 1000 Genomes Project database (1KGP) and RefSeq database.

Databases	No. of Variants	Type of Variants
Rare variant (<0.01)	3,048,219 (15.08%)	Intergenic and intron = 97.56% Non-intergenic and intron = 2.44%
Low frequency (0.01–0.05)	1,421,767 (7.03%)	Intergenic and intron = 99.255% Non-intergenic and intron = 0.75%
Common variant (>0.05)	7,596,517 (37.57%)	Intergenic and intron = 97.50% Non-intergenic and intron = 2.50%
Missing (not found in 1KGP)	8,150,799 (40.32%)	Intergenic and intron = 98.62% Non-intergenic and intron = 1.38%

**Table 4:** Disease-associated variations in the ClinVar database (version 2023) and the 1000 Genomes Project database (1KGP).

Type of Variants	Rare	Low Frequency	Common	Missing	Total
Pathogenic	56 (33.14%)	11 (6.51%)	15 (8.88%)	87 (51.48%)	169
Likely pathogenic	18 (30.00%)	1 (1.67%)	6 (10.00%)	35 (58.33%)	60
Uncertain significance	1933 (35.27%)	43 (0.78%)	98 (1.79%)	3407 (62.16%)	5481
Likely benign	5696 (36.66%)	3392 (21.83%)	2350 (15.13%)	4099 (26.38%)	15,537
Benign	5215 (5.28%)	8539 (8.65%)	71,618 (72.57%)	13,314 (13.49%)	98,686

**Table 5:** Pathological mutations with frequency < 0.01 and missing in 1KGP with frequency > 0.05 in 80 healthy Thai databases.

Chr: Pos	Identifier	Gene Names	Condition	AF in 80 Thai
1:144915624	rs66512216	*PDE4DIP*	Hepatocellular carcinoma	0.338
1:26608812	rs2073002071	*UBXN11*	Hepatocellular carcinoma, small cell lung carcinoma, lung cancer	0.088
1:26608826	rs752317296	*UBXN11*	Hepatocellular carcinoma, lung cancer	0.154
1:26608836	rs757094832	*UBXN11*	Hepatocellular carcinoma, lung cancer	0.154
1:26608866	rs764852231	*UBXN11*	Hepatocellular carcinoma, lung cancer, small cell lung carcinoma	0.108
3:113376111	rs10606566	*USF3*	Hepatocellular carcinoma	0.117
3:113376111	rs10606566	*USF3*	Hepatocellular carcinoma	0.417, 0.117
3:195508455	rs1553873983	*MUC4*	Hepatocellular carcinoma, lung cancer	0.071
6:16327865	rs751421308	*ATXN1*	Hepatocellular carcinoma	0.050
6:16327913	rs751377396	*ATXN1*	Hepatocellular carcinoma	0.242
6:170871055	rs770128377	*TBP*	Hepatocellular carcinoma	0.313
11:5248173	rs33950507	*HBB*	Hemoglobin E/beta thalassemia disease	0.063
16:72821594	rs374416547	*ZFHX3*	Lung cancer, small cell lung carcinoma	0.071
16:72821619	rs751575363	*ZFHX3*	Lung cancer	0.283
17:28564285	rs774676466		Serotonin transporter activity	0.333
19:501702	rs1555716175	*MADCAM1*	Hepatocellular carcinoma	0.417
19:501702	rs768810399, rs1555716175	*MADCAM1*	Hepatocellular carcinoma	0.417, 0.133
19:501744	rs1555716199	*MADCAM1*	Hepatocellular carcinoma	0.650
22:46191235	rs60726084	*ATXN10*	Spinocerebellar ataxia type 10	0.088
X:146993568	rs193922936	*FMR1*	Fragile-X associated tremor/ataxia syndrome	0.250

**Table 6:** All hiconfdenovo of the 40 Thai trios.

Detail	No. of Variants
hiconfdenovo	19,710
dbSNP155	
Known variants	14,503 (73.58%)
Novel variants	5207 (26.42%)
RefSeq	
Intergenic_variant	12,910 (65.50%)
Intron_variant	6453 (32.74%)
Non-intergenic_variant and intron_variant	347 (1.76%)
ClinVar	
Pathogenic	1
Likely pathogenic	0
Uncertain significance	6
Likely benign	4
Benign	18

**Table 7:** De novo variants in the ClinVar database.

Chr: Pos	Gene Names	Sequence Ontology	Effect	Conditions
Pathogenic variant				
11:65829404	*SF3B2*	stop gained	LoF	Craniofacial microsomia
Uncertain significance variants				
2:62067418	*FAM161A*	missense_variant	Missense	Retinitis pigmentosa
2:63712067	*WDPCP*	missense_variant	Missense	Bardet–Biedl syndrome
3:113664291	*GRAMD1C*	missense_variant	Missense	Inborn genetic diseases
12:104379507	*TDG*	frameshift_variant	LoF	Hereditary breast–ovarian cancer syndrome
16:28950575	*CD19*	3_prime_UTR_variant	Other	Common variable immune deficiency
16:89383444	*ANKRD11*	5_prime_UTR_variant	Other	KBG syndrome

## Data Availability

The sequencing data generated in this study are part of the Genomic Thailand database. Due to institutional agreements and data-sharing policies, the entire dataset, including raw sequencing files, is not openly accessible.

## Supplementary Materials

The following supporting information can be downloaded at https://www.mdpi.com/article/10.3390/biology14030301/s1; Figure S1: The geographical distribution of the participants in Thailand; Figure S2: The overview of the range of quality values across all bases at each position in the FastQ file; Figure S3: Histogram of read depth.; Figure S4: The number of transitions per number of transversions; Figure S5: The parent age with de novo mutations.; Table S1: Pathogenic mutation in the ClinVar database with the rare and missing variants in the Thai population.

## Abbreviations

The following abbreviations are used in this manuscript:

WGS	Whole-genome sequencing
WES	Whole-exome sequencing
DNMs	De novo mutations
gVCF	Genomic variant call format
VCF	Variant call format
SNV	Single nucleotide variant
INDEL	Insertion and deletion
INS	Insertion
MNV	Multi-nucleotide variants
1KGP	1000 Genomes Project database
A	Adenine
T	Thymine
C	Cytosine
G	Guanine
AF	Allele Frequencies
Chr: Pos	Chromosome and positions
rsID	Reference SNP cluster ID.
LoF	loss-of-function
km^2^	square kilometer
mL	Milliliter
SD	Standard deviation
CFM	Craniofacial microsomia
