# Supplementary material for: Detection of Genetic Variants in Thai Population by Trio-Based Whole-Genome Sequencing Study
Source: Biology (Basel). 2025 Mar 17;14(3):301. doi: 10.3390/biology14030301 (PMC11940159; doi:10.3390/biology14030301)
Supplement: Supplementary file 1 [file biology-14-00301-s001.zip › biology-3485643-supplementary.pdf]

Supplementary Materials

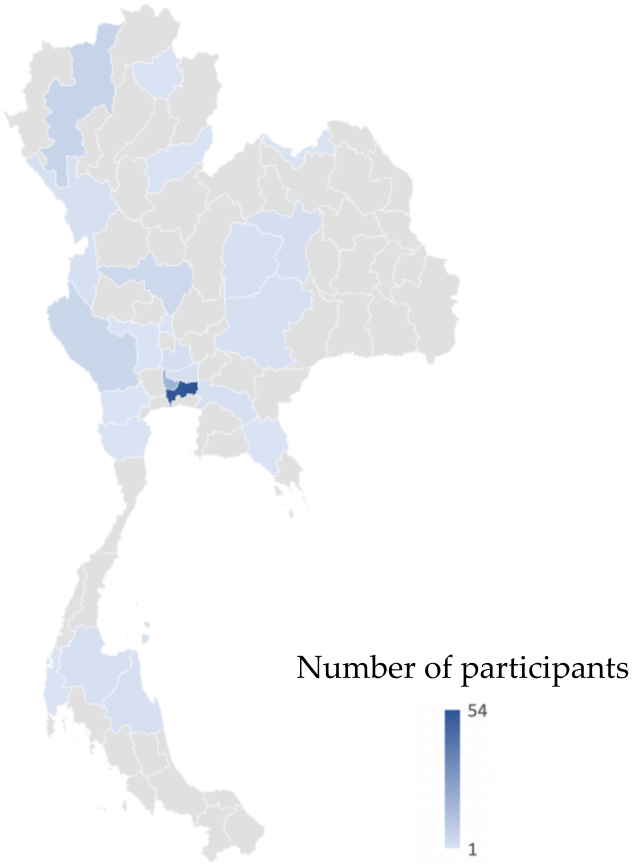

Figure S1. The geographical distribution of the participants in Thailand, a crucial aspect that underlines the relevance of our study to the Thai population.

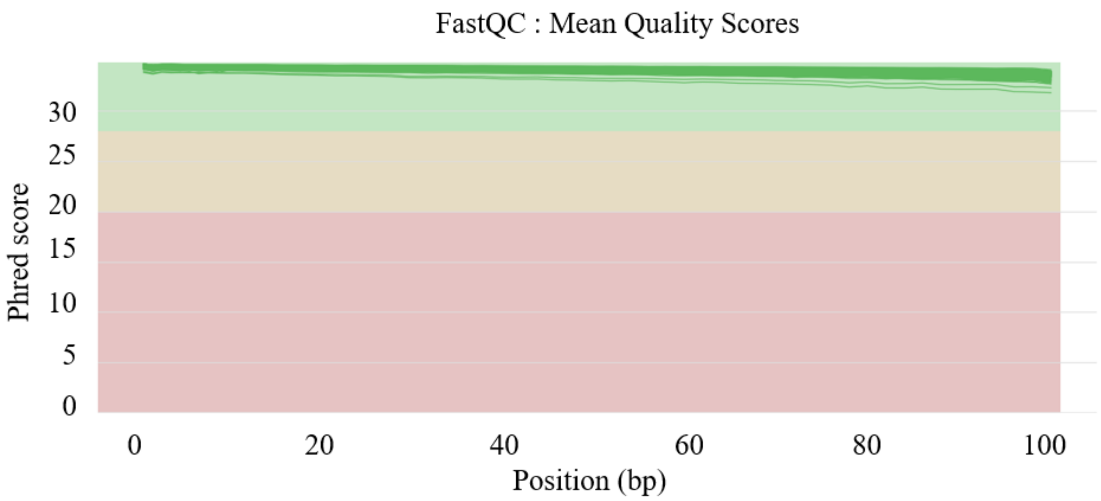

Figure S2. Overview of the range of quality values across all bases at each position in the FastQ file.

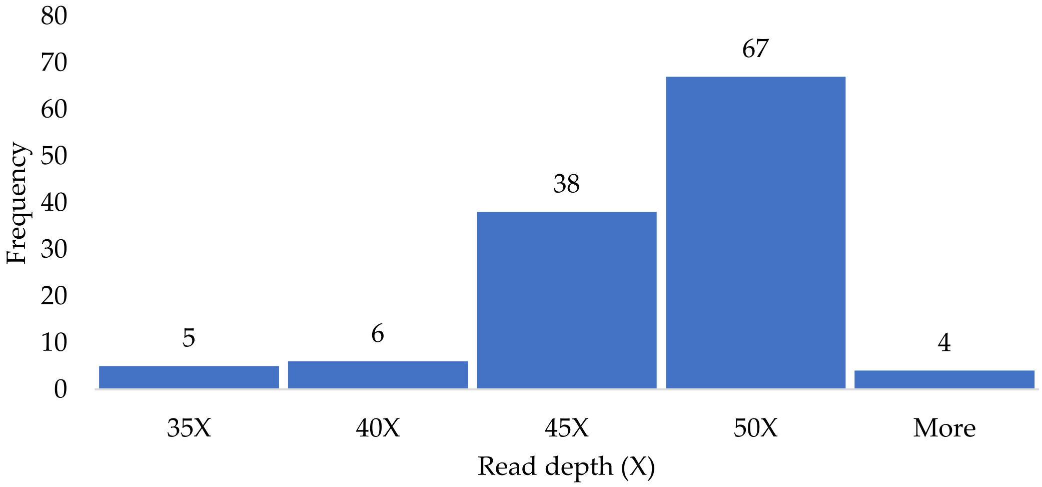

Figure S3. Histogram of read depth, a critical factor in ensuring the quality of our data. The read depth of the mapped BAM files was calculated with the 'samtool depth' function of SAMtools v1.7. The average read depth was 45.2X (minimum 32.1X and maximum 51.8X). The y-axis represents the number of samples, and the x-axis represents the read coverage interval. 90.8 percent of the sample showed a high read coverage depth (more than 40X).

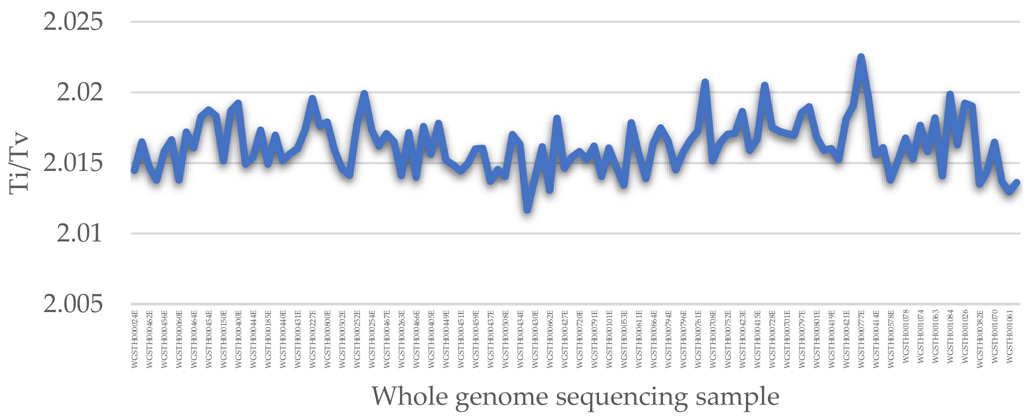

Figure S4. Number of transitions per number of transversions. After the pipeline has been finished and variants have been called, it still needs to determine whether the variants are reasonable-looking. The TiTv Ratio, the ratio of transition (Ti) to transversion (Tv) SNPs, plays a crucial role in this determination, which in whole genome sequencing should be in the range of 2.0-2.1 [22]. Figure S4 represented the number of transitions per number of transversions in 120 VCF from whole genome sequencing with an average of 2.016.

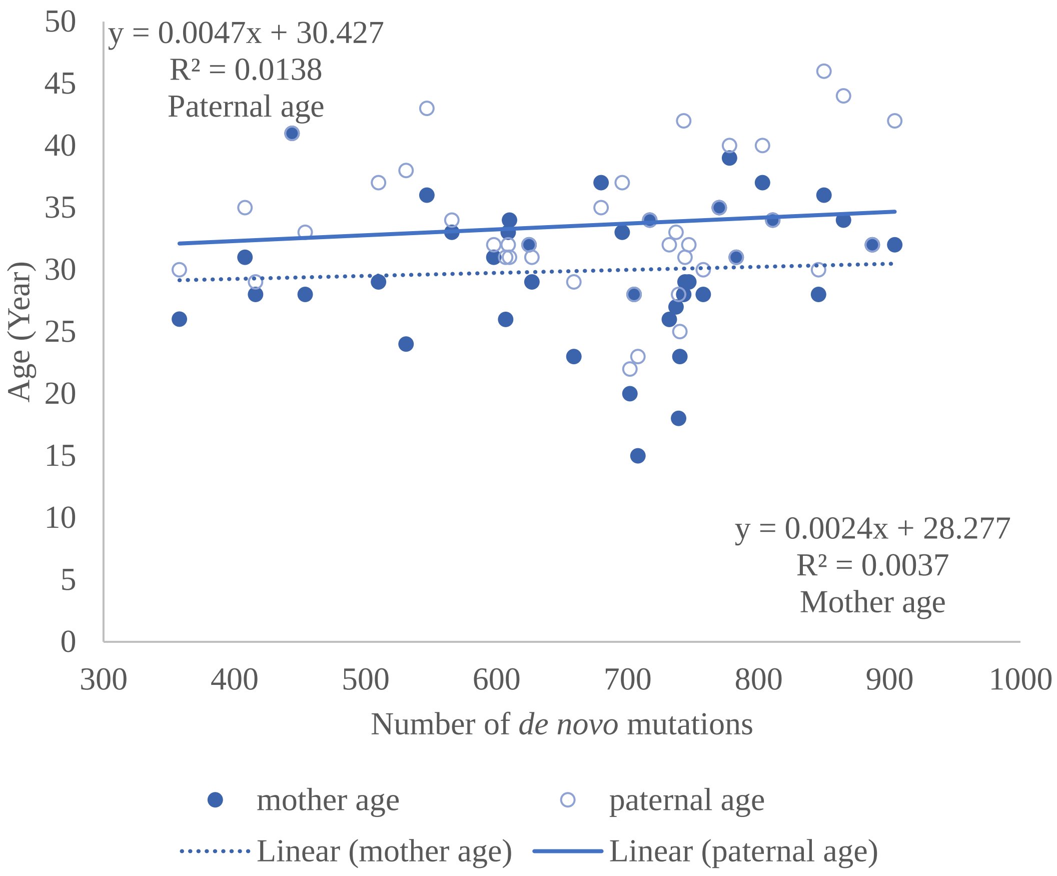

Figure S5. Parent age with *de novo* mutations.

Table S1. Pathogenic mutation in ClinVar database with rare and missing variants in the Thai population.

No.	Chr: Pos	Identifier	Gene Names	Sequence Ontology	Conditions	AF in 1KGP
1	1:978577	rs200373411	*AGRN*	intron_variant	Congenital myasthenic syndrome	0.0018
2	1:15767073	rs515726209	*CTRC*	missense_variant	Hereditary pancreatitis	0.0008
3	1:16375584	rs751608665	*CLCNKB*	frameshift_variant	Bartter disease type 3	
4	1:20972133	rs28940285	*PINK1*	missense_variant	Autosomal recessive early-onset Parkinson's disease	
5	1:26608812	rs2073002071	*UBXN11*	inframe_deletion	Hepatocellular carcinoma, Small cell lung carcinoma, Lung cancer	
6	1:26608820	rs66614970	*UBXN11*	inframe_insertion	Hepatocellular carcinoma, Lung cancer	
7	1:26608820	rs66614970	*UBXN11*	inframe_deletion	Hepatocellular carcinoma, Lung cancer	
8	1:26608826	rs752317296	*UBXN11*	frameshift_variant	Hepatocellular carcinoma, Lung cancer	
9	1:26608836	rs757094832	*UBXN11*	frameshift_variant	Hepatocellular carcinoma, Lung cancer	
10	1:26608866	rs764852231	*UBXN11*	frameshift_variant	Hepatocellular carcinoma, Lung cancer, Small cell lung carcinoma	
11	1:26608878	rs140364749	*UBXN11*	inframe_deletion	Hepatocellular carcinoma, Small cell lung carcinoma, Lung cancer	
12	1:54371889	rs201420507	*DIO1*	missense_variant	Thyroid hormone metabolism	0.0048
13	1:91404897	rs387907109	*ZNF644*	missense_variant	Myopia	
14	1:94512566	rs61749446	*ABCA4*	missense_variant	Retinal dystrophy	0.0002
15	1:98293683	rs189768576	*DPYD*	stop_gained	Dihydropyrimidine dehydrogenase deficiency	0.0006
16	1:120293450	rs372079931	*HMGCS2*	missense_variant	3-hydroxy-3-methylglutaryl-CoA synthase deficiency	
17	1:144915624	rs66512216	*PDE4DIP*	frameshift_variant	Hepatocellular carcinoma	
18	1:152275298	rs146466242	*FLG*	stop_gained	Ichthyosis Vulgaris	0.0040
19	1:161192707	rs771259264	*APOA2*	splice_donor_variant	Apolipoprotein A-II Deficiency	
20	2:1500419	rs770781635	*TPO*	frameshift_variant	Deficiency of iodide peroxidase	
21	2:43452021	rs373995498	*ZFP36L2*	missense_variant	Oocyte maturation defect	0.0002
22	2:69633155	rs1354126704	*NFU1*	missense_variant	Multiple mitochondrial dysfunction syndrome	
23	2:99013339	rs201782746	*CNGA3*	missense_variant	Retinal dystrophy	
24	2:113819812	rs139497891	*IL36RN*	missense_variant	Generalized pustular psoriasis	0.0008
25	2:128183690	rs146922325	*PROC*	missense_variant	Thrombophilia due to protein C deficiency	0.0040
26	2:128183697	rs199469469	*PROC*	inframe_deletion	Thrombophilia due to protein C deficiency	0.0008
27	2:182403965	rs1026405158	*CERKL*	frameshift_variant	Retinitis pigmentosa	
28	2:197531519	rs376590781	*CCDC150*	frameshift_variant	Hepatocellular carcinoma	
29	2:197531519	rs376590781	*CCDC150*	frameshift_variant	Hepatocellular carcinoma	
30	2:202494451	rs793888505	*TMEM237*	splice_donor_variant	Joubert syndrome	
31	2:219679419	rs200883871	*CYP27A1*	missense_variant	Cholestanol storage disease	0.0004
32	2:233407740	rs767503038	*CHRNG*	frameshift_variant	Lethal multiple pterygium syndrome	
33	2:234676872	rs34946978	*UGT1A1,UGT1A3, UGT1A4,UGT1A5, UGT1A6,UGT1A7,*	missense_variant	Hyperbilirubinemia	0.0022

No.	Chr: Pos	Identifier	Gene Names	Sequence Ontology	Conditions	AF in 1KGP
			UGT1A8,UGT1A9, UGT1A10			
34	3:3188173	rs370011798	*TRNT1*	missense_variant	Congenital sideroblastic anemia-B-cell immunodeficiency-periodic fever-developmental delay syndrome	
35	3:81584455	rs772802187	*GBE1*	missense_variant	Glycogen storage disease IV	
36	3:113376111	rs10606566	*USF3*	inframe_insertion	Hepatocellular carcinoma	
37	3:113376111	rs10606566	*USF3*	inframe_deletion	Hepatocellular carcinoma	
38	3:113376111	rs10606566	*USF3*	inframe_deletion	Hepatocellular carcinoma	
39	3:121713035	rs142746163	*ILDR1*	stop_gained	Hearing impairment	0.0020
40	3:132384674	rs532178791	*UBA5*	initiator_codon_variant	Developmental and epileptic encephalopathy	0.0006
41	3:182804537	rs1163620394	*MCCC1*	stop_gained	3-methylcrotonyl-CoA carboxylase 1 deficiency	
42	3:195508455	rs1553873983	*MUC4*	inframe_insertion	Hepatocellular carcinoma, Lung cancer	
43	4:38798417	rs56205407	*TLR1*	missense_variant	Rheumatoid arthritis	0.0016
44	4:159603421	rs121964954	*ETFDH*	missense_variant	Glutaric acidemia Iic	0.0002
45	4:187122319	rs556930061	*CYP4V2*	frameshift_variant	Bietti crystalline chorioretinal dystrophy	0.0014
46	4:187126358	rs199476197	*CYP4V2*	missense_variant	Bietti crystalline chorioretinal dystrophy	
47	5:33985177	rs984225803		intergenic_variant		
48	5:35618111	rs1391102782	*SPEF2*	frameshift_variant	Spermatogenic failure	
49	5:39341378	rs121909592	*C9*	stop_gained	Complement component 9 deficiency	0.0030
50	5:89975381		*ADGRV1*	frameshift_variant		
51	5:139422532	rs756281194	*NRG2*	inframe_deletion	Hepatocellular carcinoma	
52	5:147207583	rs148954387	*SPINK1*	splice_donor_variant	Hereditary pancreatitis	0.0008
53	5:147211355	rs191068215	*SPINK1*	5_prime_UTR_variant	Hereditary pancreatitis	0.0008
54	6:10529158	rs185805779	*GCNT2*	stop_gained	Cataract 13 with adult I phenotype	0.0010
55	6:16327865	rs751421308	*ATXN1*	inframe_insertion	Hepatocellular carcinoma	
56	6:16327865	rs751421308	*ATXN1*	inframe_deletion	Hepatocellular carcinoma	
57	6:16327913	rs751377396	*ATXN1*	inframe_deletion	Hepatocellular carcinoma	
58	6:16327916	rs193922926	*ATXN1*	inframe_insertion	Hepatocellular carcinoma	
59	6:17669692	rs200406494	*NUP153*	missense_variant	Pulmonary artery atresia	0.0004
60	6:64436538	rs184722374	*EYS*	stop_gained	Retinal dystrophy	0.0002
61	6:88239365	rs145297855	*RARS2*	missense_variant	Pontocerebellar hypoplasia type 6	0.0002
62	6:152614722	rs1562842409	*SYNE1*	splice_donor_variant	Autosomal recessive ataxia	
63	6:170871055	rs770128377	*TBP*	frameshift_variant	Hepatocellular carcinoma	
64	7:65551592	rs769017508	*ASL*	missense_variant	Argininosuccinate lyase deficiency	
65	7:80290426	rs572295823	*CD36*	frameshift_variant	Platelet-type bleeding disorder, Coronary heart disease	0.0038
66	7:95951267	rs541276426	*SLC25A13*	initiator_codon_variant	Citrin deficiency	0.0054
67	7:117149197	rs121908791	*CFTR*	splice_donor_variant	Cystic fibrosis	

No.	Chr: Pos	Identifier	Gene Names	Sequence Ontology	Conditions	AF in 1KGP
68	7:139715531	rs199422117	*TBXAS1*	missense_variant	Ghosal hematodiaphyseal syndrome	0.0010
69	8:48844057	rs546905091	*PRKDC*	intron_variant	Severe combined immunodeficiency due to DNA-PKcs deficiency	
70	9:13168408	rs376078512	*MPDZ*	stop_gained	Hydrocephalus, nonsyndromic	
71	9:36276924	rs200763627	*GNE*	stop_gained	GNE myopathy; Sialuria	0.0004
72	9:114449111	rs533026166	*SHOC1*	missense_variant	Spermatogenic failure	0.0004
73	9:123182239	rs587783387	*CDK5RAP2*	splice_acceptor_variant	Microcephaly	
74	9:131088128	rs776825296	*COQ4*	missense_variant	Neonatal encephalomyopathy-cardiomyopathy-respiratory distress syndrome	
75	10:103826855	rs1590263807	*HPS6*	frameshift_variant	Hermansky-Pudlak syndrome	
76	10:124800869	rs553730391	*ACADSB*	missense_variant	Deficiency of 2-methylbutyryl-CoA dehydrogenase	0.0002
77	11:5248173	rs33950507	*HBB*	missense_variant	Hemoglobin E/beta thalassemia disease	0.0028
78	11:10521792	rs3741040	*AMPD3*	missense_variant	Erythrocyte AMP deaminase deficiency	0.0004
79	11:62474958	rs190405606	*BSCL2*	intron_variant	Reduced delayed hypersensitivity, Breast carcinoma	0.0012
80	11:65829404		*SF3B2*	stop_gained	Craniofacial microsomia	
81	11:126215441	rs137941190	*DCPS*	missense_variant	Al-Raqad syndrome	0.0020
82	12:7053006	rs180837208	*C12orf57*	intron_variant	Aicardi-Goutieres syndrome	0.0004
83	12:40704236	rs33939927	*LRRK2*	missense_variant	Autosomal dominant Parkinson's disease	
84	12:103246714	rs76687508	*PAH*	missense_variant	Phenylketonuria	
85	13:52511774	rs767464491	*ATP7B*	missense_variant	Wilson disease	
86	13:52516618	rs541208827	*ATP7B*	missense_variant	Wilson disease	0.0004
87	13:52523908	rs121907993	*ATP7B*	missense_variant	Wilson disease	0.0002
88	13:52524205	rs786204718	*ATP7B*	missense_variant	Wilson disease	
89	13:52524252	rs121907994	*ATP7B*	missense_variant	Wilson disease	
90	13:52532498	rs137853287	*ATP7B*	frameshift_variant	Wilson disease	
91	14:23574038	rs553352307	*CIROP*	missense_variant	Heterotaxy	0.0004
92	14:65249169	rs760803657	*SPTB*	missense_variant	Elliptocytosis 3, Hereditary spherocytosis type 2	
93	15:31360239	rs777916333	*TRPM1*	frameshift_variant		
94	15:43552349	rs112292549	*TGM5*	missense_variant	Acral peeling skin syndrome	0.0014
95	15:45396177	rs774556391	*DUOX2*	missense_variant	Thyroid dyshormonogenesis	
96	15:45399648	rs180671269	*DUOX2*	stop_gained	Thyroid dyshormonogenesis	0.0006
97	15:45403974	rs201590426	*DUOX2*	missense_variant	Thyroid dyshormonogenesis	0.0006
98	15:74630995	rs775102947	*CYP11A1*	missense_variant	Congenital adrenal insufficiency with 46, XY sex reversal OR 46,XY disorder of sex development-adrenal insufficiency due to CYP11A1 deficiency	
99	15:87531320	rs79072327	*AGBL1*	splice_donor_variant	Corneal dystrophy, Fuchs endothelial	0.0052
100	16:223597	rs41464951	*HBA2*	stop_lost	Hemoglobin constant spring, Hemoglobin H disease	0.0002
101	16:3293205	rs104895097	*MEFV*	missense_variant	Familial Mediterranean fever	

No.	Chr: Pos	Identifier	Gene Names	Sequence Ontology	Conditions	AF in 1KGP
102	16:16256866	rs72653744	*ABCC6*	stop_gained	Pseudoxanthoma elasticum	
103	16:23391491	rs550424284	*SCNN1B*	splice_donor_variant		0.0002
104	16:56919275	rs200697179	*SLC12A3*	missense_variant	Familial hypokalemia-hypomagnesemia	0.0002
105	16:57935311	rs752967885	*CNGB1*	missense_variant	Retinitis pigmentosa	
106	16:70513133	rs199515460	*FCSK*	missense_variant	Congenital disorder of glycosylation with defective fucosylation	0.0002
107	16:72821594	rs374416547	*ZFHX3*	inframe_insertion	Lung cancer, Small cell lung carcinoma	
108	16:72821619	rs751575363	*ZFHX3*	inframe_deletion	Lung cancer	
109	16:72831358	rs34918837	*ZFHX3*	inframe_deletion	Lung cancer	
110	16:84188336	rs786205052	*DNAAF1*	frameshift_variant	Primary ciliary dyskinesia	
111	17:7125591	rs113994167	*ACADVL*	missense_variant	Very long-chain acyl-CoA dehydrogenase deficiency	
112	17:11502117	rs769795916	*DNAH9*	frameshift_variant	Abnormality of cardiovascular system morphology, Ciliary dyskinesia	
113	17:19284408	rs147296805	*MAPK7*	missense_variant	Scoliosis	0.0014
114	17:28564285	rs774676466		intergenic_variant	Serotonin transporter activity	
115	17:33902992	rs398123301	*PEX12*	frameshift_variant	Peroxisome biogenesis disorder 3A (Zellweger)	
116	17:42460885	rs1214448436	*ITGA2B*	missense_variant	Glanzmann thrombasthenia	
117	17:48245758	rs372210292	*SGCA*	missense_variant	Autosomal recessive limb-girdle muscular dystrophy type 2D	
118	17:78358945	rs112735431	*RNF213*	missense_variant	Moyamoya disease	0.0012
119	18:43314238	rs78937798	*SLC14A1*	splice_acceptor_variant	Jk-null variant	0.0010
120	18:61465905	rs672601344	*SERPINB7*	frameshift_variant	Palmoplantar keratoderma	
121	18:61468149	rs534014297	*SERPINB7*	frameshift_variant	Palmoplantar keratoderma, Nagashima type	0.0006
122	19:501702	rs1555716175	*MADCAM1*	inframe_insertion	Hepatocellular carcinoma	
123	19:501702	rs768810399, rs1555716175	*MADCAM1*	frameshift_variant	Hepatocellular carcinoma	
124	19:501744	rs1555716199	*MADCAM1*	inframe_insertion	Hepatocellular carcinoma	
125	19:501744	rs1555716194, rs1555716199	*MADCAM1*	frameshift_variant	Hepatocellular carcinoma	
126	19:49206817	rs1800028	*FUT2*	stop_gained	Vitamin b12 plasma level quantitative trait locus 1, Familial Otitis Media	0.0008
127	19:50982310	rs770255014	*EMC10*	frameshift_variant	Intellectual disability, Neurodevelopmental disorder with dysmorphic facies, and variable seizures	
128	20:2840713	rs367642720	*VPS16*	missense_variant	Dystonia	0.0008
129	20:62324564	rs398123017	*RTEL1*	stop_gained	Dyskeratosis congenita	
130	22:18604444	rs776983625	*TUBA8*	missense_variant	Macrothrombocytopenia	
131	22:20920814		*MED15*	inframe_deletion	Hepatocellular carcinoma	
132	22:29130458	rs1601851875	*CHEK2*	frameshift_variant	Familial cancer of the breast	
133	22:46191235	rs60726084	*ATXN10*	intron_variant	Spinocerebellar ataxia type 10	

No.	Chr: Pos	Identifier	Gene Names	Sequence Ontology	Conditions	AF in 1KGP
134	22:46191235	rs60726084	*ATXN10*	intron_variant	Spinocerebellar ataxia type 10	
135	22:46191235	rs60726084	*ATXN10*	intron_variant	Spinocerebellar ataxia type 10	
136	22:50962802		*NCAPH2, SCO2*	frameshift_variant		
137	X:38260674	rs72556284	*OTC*	missense_variant	Ornithine carbamoyltransferase deficiency	
138	X:66765159	rs3032358	*AR*	inframe_deletion	Hepatocellular carcinoma, Androgen resistance syndrome, Kennedy disease	
139	X:66937337	rs137852571	*AR*	missense_variant	Prostate cancer	0.0005
140	X:146993568	rs193922936	*FMR1*	5_prime_UTR_variant	Fragile X-associated tremor/ataxia syndrome, Premature ovarian failure	
141	X:153760484	rs72554665	*G6PD*	missense_variant	G6PD	0.0016
142	X:153760605	rs398123546	*G6PD*	missense_variant	Anemia	0.0005
143	X:154159225	rs782318401	*F8*	missense_variant	Thrombophilia	0.0003
